# Inhibition of Polo-like kinase 2 ameliorates pathogenesis in Alzheimer’s disease model mice

**DOI:** 10.1371/journal.pone.0219691

**Published:** 2019-07-15

**Authors:** Ji Soo Lee, Yeunkum Lee, Emily A. André, Kea Joo Lee, Thien Nguyen, Yang Feng, Nuo Jia, Brent T. Harris, Mark P. Burns, Daniel T. S. Pak

**Affiliations:** 1 Department of Pharmacology and Physiology, Georgetown University Medical Center, Washington, DC, United States of America; 2 Department of Neurology, Georgetown University Medical Center, Washington, DC, United States of America; 3 Department of Neuroscience, Georgetown University Medical Center, Washington, DC, United States of America; Nathan S Kline Institute, UNITED STATES

## Abstract

Alzheimer disease (AD) is a neurodegenerative disorder characterized by pathological hallmarks of neurofibrillary tangles and amyloid plaques. The plaques are formed by aggregation and accumulation of amyloid β (Aβ), a cleavage fragment of amyloid precursor protein (APP). Enhanced neuronal activity and seizure events are frequently observed in AD, and elevated synaptic activity promotes Aβ production. However, the mechanisms that link synaptic hyperactivity to APP processing and AD pathogenesis are not well understood. We previously found that Polo-like kinase 2 (Plk2), a homeostatic repressor of neuronal overexcitation, promotes APP β-processing *in vitro*. Here, we report that Plk2 stimulates Aβ production *in vivo*, and that Plk2 levels are elevated in a spatiotemporally regulated manner in brains of AD mouse models and human AD patients. Genetic disruption of Plk2 kinase function reduces plaque deposits and activity-dependent Aβ production. Furthermore, pharmacological Plk2 inhibition hinders Aβ formation, synapse loss, and memory decline in an AD mouse model. Thus, Plk2 links synaptic overactivity to APP β-processing, Aβ production, and disease-relevant phenotypes *in vivo*, suggesting that Plk2 may be a potential target for AD therapeutics.

## Introduction

Alzheimer’s Disease (AD) is a neurodegenerative disorder characterized by progressive cognitive decline and memory deficits [1]. According to the amyloid hypothesis, an abnormal accumulation of amyloid β (Aβ) initiates pathogenesis, based on observations that aggregated Aβ is the primary constitutent of hallmark senile plaques [2,3], and familial AD (FAD) mutations that cause early-onset disease promote Aβ production and aggregation [4–7]. Furthermore, memory loss can be induced in wild-type rodents by injection of soluble Aβ oligomers, suggesting this conformation instigates neuronal dysfunction [8–10]. Aβ is derived from sequential cleavage of amyloid precursor protein (APP) by β- and γ-secretase [3]. This amyloidogenic processing is considered a major pathological mechanism and thus intensively studied for therapeutics in AD. However, mechanisms that exert physiological control over the rate of APP cleavage and Aβ production are still unclear.

Development of AD mouse models has contributed significantly to the elucidation of underlying cognitive impairment mechanisms. These models are genetically engineered to recapitulate the pathological hallmarks of AD, β-amyloid plaques and/or neurofibrillary tangles. Although none of the mouse models fully represent the range of phenotypes found in AD patients, they show similar changes associated with disease progression and cognitive decline [11,12]. For example, APP-SwDI transgenic mice overexpressing human APP bearing three distinct FAD mutations demonstrate significantly enhanced Aβ production, plaque deposits, and cognitive impairments [13–15]. Moreover, 5XFAD transgenic mice, with 5 distinct FAD mutations in both APP and presenilin (the enzymatic subunit of γ-secretase), show accelerated plaque formation and memory decline as well as higher levels of Aβ42, a more pathogenic form of Aβ, relative to the less aggregating Aβ40 form [12,16]. These AD mouse models have been widely used to evaluate potential therapeutic candidates to counteract Aβ-mediated pathogenic processes.

A notable characteristic of many APP transgenic mouse models is abnormal epileptiform activity as assessed by electroencephalogram (EEG) recordings [17–19], which is a feature also commonly observed in AD patients [20]. Moreover, both AD patients and AD mouse models have a high incidence of seizure, and heightened neuronal activity promotes Aβ production *in vitro* and *in vivo* [21–23]. These observations suggest that neuronal hyperactivity may drive excessive Aβ formation during AD. During seizure-like activity, expression of the activity-inducible gene Polo-like kinase (Plk) 2 is robustly upregulated. For instance, Plk2 is markedly induced in rats given acute and chronic electroconvulsive shock and in a rat model of hypoxia-induced neonatal seizure [24,25]. Plk2 is a negative feedback homeostatic regulator that acts by triggering synaptic depression and dendritic spine elimination via multiple pathways to reduce synaptic hyperactivity [26–29]. Previously, we reported the *in vitro* finding that Plk2 kinase function promotes activity-dependent APP β-processing and Aβ production by directly binding to and phosphorylating threonine (668) and serine (675) of synaptic APP [30], and may be one of multiple kinases that contributes to hyperphosphorylation of APP in human AD brain [31,32]. Indeed, Plk2 expression is elevated in AD brain tissue [33] and genetic variants of Plk2 have been associated with AD [34]. These observations suggest Plk2 may be induced by neuronal hyperactivity during AD and contribute to APP amyloidogenic processing via phosphorylation.

Here, we show that brains of AD mice and post-mortem AD patients displayed spatiotemporally regulated elevation in Plk2 levels. Genetically inhibiting Plk2 kinase function reduced Aβ plaque formation and activity-induced Aβ production in the forebrains of AD mouse models. Furthermore, pharmacological inhibition of Plk2 also reduced Aβ production, synapse loss, and memory impairments in the AD model mice. These *in vivo* findings utilizing a combination of genetic and pharmacological approaches implicate Plk2 as a potential regulator of Aβ formation, Aβ-mediated synapse loss, and subsequent cognitive decline.

## Materials and methods

### Ethics statement

All animal methods were performed in strict accordance with guidelines and regulations of the Georgetown University Institutional Animal Care and Use Committee (IACUC) and the Guide for the Care and Use of Laboratory Animals of the National Institutes of Health, and all experimental protocols were approved by the Georgetown IACUC (protocol numbers 2016–1144 and 2016–1145). All efforts were made to minimize suffering including flow-rate controlled CO_2_ for euthanasia. For human subjects, Georgetown University Medical Center does not require IRB approval or informed consent on de-identified post-mortem human brain blocks obtained from tissue banks.

### Cell cultures

Hippocampal neurons were prepared from E18 rat embryos. Coverslips were submerged in nitric acid for 2 days and then in water overnight, followed by overnight baking at 200°C. The coverslips were coated with poly-D-lysine overnight and then laminin over 5 hours in 12-well plates. Neurobasal media (Invitrogen) supplemented with SM1 (Stemcell), 0.5 mM glutamine and 12.5 μM glutamate was added in the plates at least an hour before plating neurons. Neurons were plated at ~75,000 cells/well (~150 cells/mm^2^) and grown at 37°C in a humidified chamber with 95% O_2_/5% CO_2_.

### Antibodies

The following antibodies were purchased from the indicated suppliers and used at the indicated dilutions: APP-N (Sigma A8967, ICC 1:400), Y188 (OriGene, IB:1:1000~1:7000); sAPPβ (Immuno-Biological Laboratories, IB 1:500); sAPPα (Immuno-Biological Laboratories 2B3, IB 1:100); 6E10 (Covance, IHC 1:500, IB 1:1000); Plk2 C-terminal C-18 and H-90 (Santa Cruz, IHC 1:200); synaptophysin (Sigma, IB 1:500); β-actin (Sigma, IB 1:5000); anti-mouse PSD-95 (NeuroMAb, ICC 1:200). AlexaFluor-488 and AlexaFluor-555 (Invitrogen, ICC 1:200~400) were secondary antibodies used for immunocytochemistry.

### Inhibitors

The following inhibitors were used: protease inhibitor and phosphatase inhibitor I and II (Sigma), volasertib (BI6727) (Selleckchem). Picrotoxin (PTX) (Acros) was freshly prepared as a 10 mM stock in 0.1 M NaOH and treated at 25 μM final concentration in hippocampal cultured neurons for 18–20 hours.

### Immunocytochemistry

Primary hippocampal cultured neurons were fixed in ice-cold methanol for 7 min followed by 1% paraformaldehyde for 5 min, and incubated with primary antibody in GDB buffer (0.1% gelatin (wt/vol), 0.3% Triton X-100 (vol/vol), and 450 mM NaCl in phosphate buffered saline (PBS)). Cells were treated with Alexa 488- and Alexa 555-tagged secondary antibody (Invitrogen) in GDB buffer.

### Immunoblotting

Forebrain samples of 5xFAD mice were first dissolved in 5 M guanidine HCl/50mM Tris HCl for ELISAs, and then processed with SDS-PAGE Sample Prep Kit (Thermo Scientific Pierce) to remove guanidine HCl according to the manufacturer’s instructions. Proteins were diluted in Laemmli sample buffer and separated by Tris-glycine SDS-PAGE. Proteins were transferred to 0.45 μm pore size nitrocellulose and detected with enhanced chemiluminescence (Pierce) following incubation of HRP-linked secondary antibody. To obtain better resolution and retention of low molecular weight APP-CTF species, protein samples were separated on a 15% Tris-tricine SDS-PAGE gel and transferred to 0.2 μm pore size nitrocellulose.

### Animals

Wild-type C57BL/6J, APP-SwDI^+/+^ (*C57BL/6-Tg*(*Thy1-APPSwDutIowa*) *BWevn/Mmjax* (stock #007027), and 5XFAD (*B6*.*Cg-Tg(APPSwFlLon*,PSEN1*M146L*L286V)6799Vas/Mmjax, stock #008730) mice were purchased from Jackson Laboratory. To generate Plk2-KD mice expressing dominant negative (DN)-Plk2 in the postnatal forebrain, a DN-Plk2 transgene construct containing 8.5 kb of CaMKIIα promoter, myc epitope-tagged DN-Plk2 (full-length rat Plk2 containing K108M inactivating point mutation within the kinase catalytic site), and SV40 intron/polyA were created previously [28]. The selected clone was microinjected into pronuclei, and transgenic mice were backcrossed to C57BL/6 wild-type mice >10 generations to generate a strain congenic with C57BL/6. PCR of genomic DNA was performed from mouse tail for genotyping. Husbandry of the animals was provided by Georgetown University Animal Facility. Light/dark cycles were 12 hrs (6 am-6 pm light) and the maximum number maintained was four mice per cage. APP-SwDI mice were initially used to see primarily changes in beta-amyloid production and plaque formation. Subsequently, a 5XFAD mouse model with aggressive and fast-developing pathology was employed that display more prominent synapse loss and memory deficits. For 5XFAD mice, we used only male mice to reduce the variability of behavioral assays. For all other strains, equal numbers of male and female mice were used. Mice with ages 2–3 months apart were binned and animals were randomly assigned numbers by another investigator not involved in the study, and these numbers used to randomly assign animals to experimental groups as well as to treatment conditions during experimentation. All analysis was performed blinded to condition as well.

### Tissue staining

Fresh frozen brain cryosections (20 μm-thick) were fixed with 4% paraformaldehyde for 10 min, attached to gelatin coated slides and stained with 1% thioflavin S or human Aβ antibody 6E10 (1:100~500) to detect amyloid plaques. Alternatively, mouse brains were perfusion-fixed with 4% paraformaldehyde and immunohistochemistry performed on free-floating sections (20 or 40 μm-thick) using two independent Plk2 antibodies C-18 (C-terminal epitope) and H-90 (N-terminal epitope) (Santa Cruz Biotechnology, 1:100).

### Human brain samples and immunohistochemistry

Paraffin embedded blocks of post-mortem human brain samples were obtained from the Georgetown Brain Bank. The blocks were from 6 cases of AD (5 cases in cerebellar samples due to loss of one case) and 5 cases of non-demented controls. Temporal and cerebellar regions were obtained from the same individual. Blocks were cut in 5 μm-thick sections in the Histopathology and Tissue Shared Resource (HTSR) of Georgetown Lombardi Comprehensive Cancer Center. Sections were deparaffinized with xylene and rehydrated with gradient concentrations of ethanol. Following antigen retrieval in 10 mM citrate buffer (pH 6.0–6.5) and blocking of endogenous peroxidase activity with 1% H_2_O_2,_ blocking was done in 5% antiserum (5% BSA and horse serum in TBS). Sections were incubated with Plk2 antibody (H-90, 1:50) in 2% antiserum (2% BSA and horse serum in TBS) at 4°C overnight. The next day, sections were incubated with biotinylated goat anti-rabbit for 1 hour and Avidin-biotin complex (ABC) reagent (VECTASTAIN Elite ABC Kit; Vector laboratories) for 30 min at room temperature. 3,3’-Diaminobenzidine (DAB) peroxidase substrate (Vector laboratories) was incubated for 2 min., and then dehydration with ascending ethanol gradients and xylene and mounting with Permount (Fisher Scientific) were performed. Detailed procedures have been described [33]. Adjacent sections of all cases were also used in parallel for Mayer’s hematoxylin staining (Fisher Scientific) as described in the manufacturer’s method.

### In vivo activity modulation and pharmacological Plk inhibition

For chronic pharmacologic elevation of activity *in vivo* [35], adult 16–20 month old mice (C57BL/6, Plk2-KD[28], APP-SwDI+/-, and APP-SwDI+/-;Plk2-KD+/-) were administered a daily intraperitoneal injection for 1 week with saline or sub-seizure dose of picrotoxin (PTX, 1 mg/kg, in a volume of 10.0 ml/kg, i.p. [35]). PTX was dissolved in sterile phosphate buffered saline and prepared freshly for the daily injection. For inhibition of Plk activity *in vivo* [36], volasertib (BI6727) was dissolved in hydrochloric acid (0.1 N) and diluted with 0.9% NaCl. Male 5XFAD or WT mice at 2–4 months of age were given saline or volasertib (70 mg/kg, in a volume of 10.0 ml/kg, via oral gavage [36]) once per week for 1–3 months.

### Aβ ELISA

Mouse forebrains were homogenized in cold 5 M guanidine HCl and 50 mM Tris pH 8.0 with protease inhibitors and mixed for 3–4 hours. High concentration of guanidine HCl was used a chaotropic agent to extract insoluble Aβ and final concentration was below 0.1M [37–39]. The sample was diluted with cold reaction buffer (PBS with 5% BSA and 0.03% Tween-20) with protease inhibitors and centrifuged at 16,000 x g for 20 min, and the supernatant saved for assay. Extracts were analyzed for mouse Aβ40 as well as human Aβ40 and Aβ42 using species-specific sandwich ELISA (Invitrogen) as described [40].

### Novel object recognition test

The novel object recognition test was performed with the no-habituation session as reported [41]. Two identical objects were placed 10 cm apart and 10 cm away from the walls (sides and front) in a rectangular arena (dimensions in 40 cm x 33 cm x 20 cm). The subject mouse was placed in the arena, its head positioned toward the side opposite the objects, and allowed to explore the cage (training). After 24 hours we performed the test session to assess recognition memory, which was done in the same manner as the training except that we replaced one of the objects with a novel object. The position of the objects (left or right) and the order of trials among groups were randomized. Each trial was completed when the mouse explored between the two objects for 20 seconds, which was recorded with a stopwatch. In order to remove olfactory cues, the objects and the arena were thoroughly cleaned with 70% ethanol after each trial. The behavior of the mice was recorded by an overhead camera linked to a computer with an automated tracking system (ANYmaze 4.7 software, Stoelting Co). We used only male mice in order to avoid behavioral variances caused by difference in sex.

### Quantification and image analysis

Images were obtained using an Axiovert 200M (Zeiss) epifluorescence microscope for cultured neurons, LSM510 Meta confocal microscope (Zeiss) for brain sections (13–20 month-old), and upright microscope BX53 (Olympus) for human brain sections. The image in S1K Fig was obtained from multiple Z-sections of the confocal microscope to show morphology of cells. Metamorph and ImageJ software was used for image analysis. From cultured neuron immunocytochemistry, integrated intensity was measured in 20–30 μm dendritic segments from proximal regions (within 40 μm distance from soma) using Metamorph software. From animal and human brain sections, integrated intensity of Plk2, number of neurons with Plk2 immunoreactivity, and area of amyloid plaques detected by thioflavin S or 6E10 immunoreactivity were measured using ImageJ software, using regions of interest with equal surface area for each comparison group. Samples were processed under the same conditions in parallel to minimize handling and incubation differences, and image acquisition used consistent exposure times to prevent differences due to camera settings. We focused on clusters and cells by removing the contribution of background signal, which was accomplished by measuring and subtracting the lowest intensity region from each brain section. Intensity of plaques or Plk2 clusters was then measured using the background subtracted images. For all image analysis we were blinded to condition in order to prevent bias in quantification.

### Statistical analysis

All values were expressed as mean±SEM of at least duplicate experiments. Sample number represents the number of sections, regions, neurons, animals, or human cases as indicated in the figure legends. For analysis of immunofluorescent intensity in cultured hippocampal neurons, 3 dendritic segments from each neuron were measured and averaged per neuron. To quantify Plk2 intensity in post-mortem human samples, three sections per case were used for immunohistochemistry, and hematoxylin staining with adjacent sections was done in order to normalize Plk2 intensity by the number of cells. For the quantification of plaque areas in 13–16 month-old mice, regions between retrosplenial and primary somatosensory cortex were used; for hippocampus, we used dorsal regions as this area is more associated with cognitive functions, rather than ventral which is more associated with emotional memory. From each mouse, 2–3 sections were taken in each mouse and 6–10 random areas sampled from each section. The values from multiple sections and areas were averaged to represent each mouse. Two-tailed unpaired Student’s t-tests were used for comparisons between two independent groups, and one-way analysis of variance (ANOVA) was used for multiple group comparisons with Tukey’s *post hoc* test. Statistically significant differences were determined at *P*<0.05. All experiments were performed blind with respect to condition to avoid sampling bias.

## Results

### Elevated Plk2 levels in APP-SwDI mice and in human AD brain

**Fig 1:**
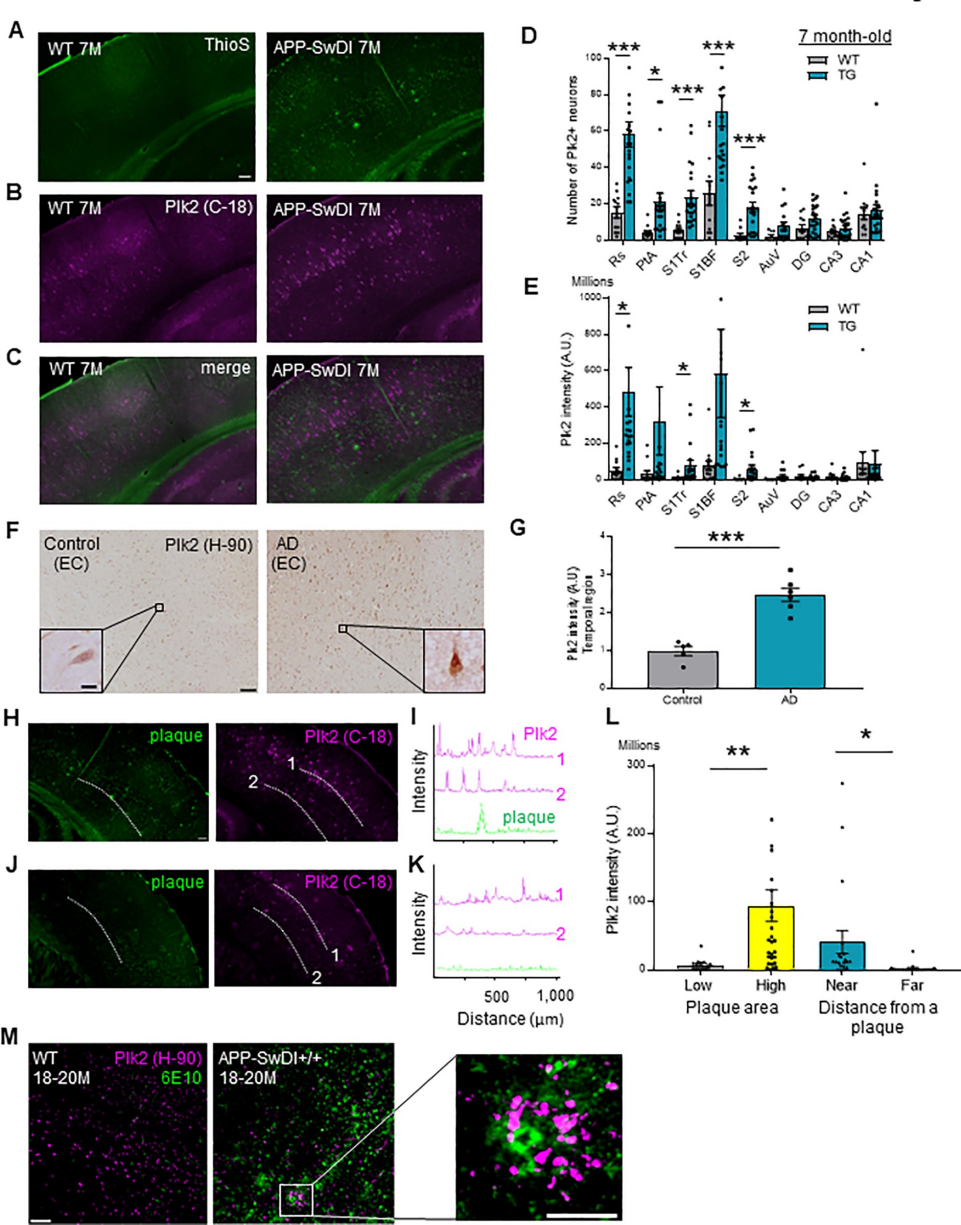
Plk2 upregulation in APP-SwDI and AD brain. (**A-C**) Representative sections of primary somatosensory cortex from 7-month-old WT C57BL/6 or APP-SwDI^*+/+*^ mice as indicated, stained with thioflavin S (**A**) and immunolabeled with Plk2 antibody (C-terminal epitope) C-18 (**B**), with merge in (**C**). Scale, 100 μm. (**D, E**) Number of Plk2 expressing neurons (Rs: *t* (31) = 4.289, *p* = 0.0002; PtA: *t* (31) = 2.699, *p* = 0.0112; S1Tr: t (31) = 3.811, *p* = 0.0006; S1BF: *t* (31) = 3.689, *p* = 0.0009; S2: *t* (29) = 3.805, *p* = 0.0007) (**D**) and Plk2 integrated intensity (Rs: *t* (29) = 2.551, *p* = 0.0163; S1Tr: *t* (30) = 2.112, *p* = 0.0431; S2: *t* (27) = 2.081, *p* = 0.0471) (**E**) at 7 months in WT and APP-SwDI^*+/+*^ transgenic (TG) mice (n = 9–22 regions from 6 animals); all statistical comparisons are between WT and TG per region (see **S1D Fig** for schematic of regions analyzed). (**F**) Representative sections of post-mortem human entorhinal cortex (EC) from non-demented control and age matched AD patients as indicated, immunolabeled with Plk2 antibody H-90. Scale bars, 100 μm (wide field), 10 μm (inset). (**G**) Quantification of Plk2 intensity in temporal regions (hippocampus and EC) (n = 5 cases for control and n = 6 for AD; *t* (9) = 6.491, *p* = 0.0001). (**H, J**) Representative thioflavin S and Plk2 staining in plaque-rich (**H**) and plaque-lacking (**J**) areas. (**I, K**) Line scans of indicated lamina from **H, J**. (**L**) Left bars, Plk2 integrated intensity per 400 μm-width cortical subdivision region of interest containing <1000 μm^2^ visible plaque area (low: n = 16 regions from 6 animals per genotype) or >1000 μm^2^ visible plaque area (high: n = 29; low vs high: *t* (43) = 2.737, *p* = 0.0090). Right bars, Plk2 intensity measured <100 μm (near; n = 20) and >400 μm (far; n = 17; near vs far: *t* (35) = 2.119, *p* = 0.0412) from plaques. (**M**) Representative sections of primary somatosensory cortex in WT and APP-SwDI*+/+* mice at 18–20 months co-immunolabeled with 6E10 for plaque staining and Plk2 H-90. Right, magnified view of boxed area. Scale bars, 50 μm (wide field), 10 μm (higher magnification). ***p < 0.001, **p < 0.01, *p < 0.05; two-tailed unpaired Student’s t-test. Data are means± SEM. Experiments were performed in at least duplicate.

To address the relation between APP amyloidogenic processing and Plk2 expression, we used the APP-SwDI mouse model of AD that produces visible amyloid plaques in the hippocampus by 3 months of age in homozygous animals, with further deposition in cortex and thalamus by 6 months [42]. Plaques were analyzed by both thioflavin S (thioS) staining and Aβ antibody 6E10 immunohistochemistry, which produced closely overlapping staining patterns in APP-SwDI+/+ brain (but no signal from WT) (S1A and S1C Fig). In 4-month-old APP-SwDI+/+ mice, modest plaque deposition began accumulating initially within hippocampal area CA1 and retrosplenial cortex, accompanied by elevated expression of Plk2 (S1D–S1H Fig). By 7 months, more widespread plaques were evident in APP-SwDI+/+ throughout cortex and hippocampus, with the exception that no further increase was observed in area CA1 (S1H Fig). At this older time point of 7 months, Plk2-expressing neurons dramatically increased in number and staining intensity throughout the APP-SwDI+/+ cortex compared to WT controls, but interestingly Plk2 was no longer upregulated in CA1, suggesting its expression may be restricted to areas of highly active amyloid production (Fig 1A–1E). Importantly, Plk2 upregulation was also observed in temporal regions of human AD compared to non-demented controls as previously reported [33], but not in regions unaffected in AD (cerebellum and occipital lobe) [3,43,44] (Fig 1F and 1G and S1I and S1J Fig). Plk2 expression was prominent in neurons of both rodent [25] and human brains (S1K and S1L Fig). The demographic and clinical characteristics of the samples are provided in S1 Table, which shows the pronounced neuropathological changes in AD post-mortem patient brains based on ABC score [45,46]. As a specificity control, we detected no signal with only secondary antibodies (S1M and S1N Fig).

Interestingly, Plk2 induction in APP-SwDI+/+ brain appeared to occur preferentially in the vicinity of plaques. Indeed, Plk2 immunoreactivity was sharply increased in cortical subregions containing at least 1000 μm^2^ visible plaque area, and Plk2 intensity was significantly higher near plaques (<100 μm) compared to plaque-distant regions (>400 μm) (Fig 1H–1L). Correlation of cortical Plk2 levels and plaque size was corroborated in aged APP-SwDI+/+ mice (18–20 months of age) using a second, independent Plk2 antibody (Fig 1M). Thus, Plk2 expression is elevated in human AD and APP-SwDI brains correlating with plaque load and proximity.

### Plk2 inhibition suppresses plaque formation in APP-SwDI mice

**Fig 2:**
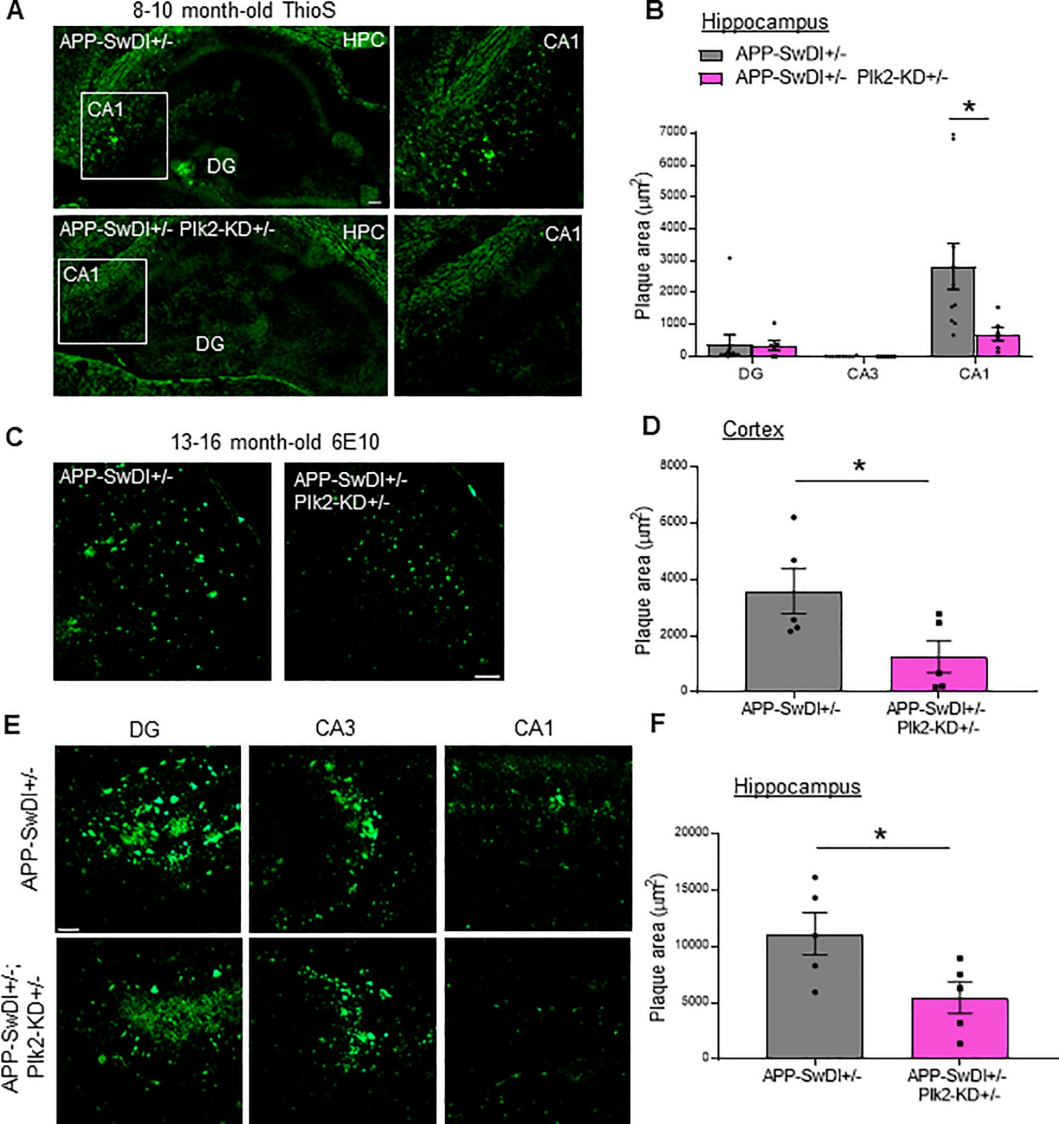
Plk2-KD inhibits plaque formation *in vivo*. (**A**) Representative APP-SwDI+/- and APP-SwDI+/-;Plk2-KD+/- thioflavin S-stained hippocampal (Hpc) cryosections. Scale, 100 μm. Enlarged view of boxed region in area CA1 is shown at right of wide-field image. (**B**) Quantification of plaque area in hippocampal subregions (n = 10 regions from 5 animals for APP-SwDI+/- and n = 6 regions from 6 animals for APP-SwDI+/-;Plk2-KD+/-; CA1: *t* (14) = 2.202, *p* = 0.0450). (**C, E**) Representative perfused brain sections in 13-16-month-old APP-SwDI+/- and APP-SwDI+/-;Plk2-KD+/- cortical (**C**) and hippocampal (**E**) areas immunolabeled with 6E10 for plaque staining. Scale, 50 μm. (**D, F**) Quantification of plaque area in (**D**) regions between retrosplenial and primary somatosensory cortex and (**F**) dorsal hippocampus (n = 5 animals per genotype; cortex: *t* (8) = 2.37, *p* = 0.0453; hippocampus: *t* (8) = 2.424, *p* = 0.0416). From each mouse, 6–10 random areas obtained from 2–3 sections were taken. The values from multiple sections and areas were averaged to represent each mouse. *p < 0.05 comparing APP-SwDI+/- and APP-SwDI+/-;Plk2-KD+/- littermates, Student’s t-test.

To investigate whether inhibition of Plk2 would affect APP amyloidogenic processing, we crossed APP-SwDI+/+ animals with previously validated transgenic mice carrying forebrain-specific expression of a Plk2-kinase-dead (KD) mutant [28]. In these transgenic mice, Plk2-KD protein expression is ~3 times that of endogenous Plk2, leading to a dominant negative impairment of normal Plk2 signaling as shown by enhanced Rap activation, increased dendritic spine density/width in CA1 area, and exaggerated contextual fear conditioning responses [28]. As a negative control for staining specificity, we confirmed that plaques were absent in Plk2-KD mice lacking APP-SwDI, as expected (S1C Fig). In APP-SwDI+/- hemizygous mice, plaques accumulated in hippocampal area CA1 at 8–10 months, but less robustly in dentate gyrus or CA3 as measured by thioS staining (Fig 2A and 2B); plaque burden overall was similar to 4-month-old APP-SwDI+/+ homozygotes. Strikingly, APP-SwDI+/-; Plk2-KD+/- double hemizygous mice at 8–10 months showed significantly decreased amyloid plaques in CA1 compared to APP-SwDI+/- littermates (Fig 2A and 2B). In older mice (13–16 months) with increased plaque burden, markedly reduced plaques were observed by 6E10 staining in APP-SwDI+/-;Plk2-KD+/- mice compared to APP-SwDI+/- littermates throughout cortex (Fig 2C and 2D) as well as hippocampus (Fig 2E and 2F). As an internal control, in these same animals we examined the thalamus, a region where the Plk2-KD transgene is not expressed [28], and found no difference in plaque formation between groups (S2A and S2B Fig).

### Inhibition of Plk2 kinase function reduces Aβ levels and activity-dependent Aβ production

**Fig 3:**
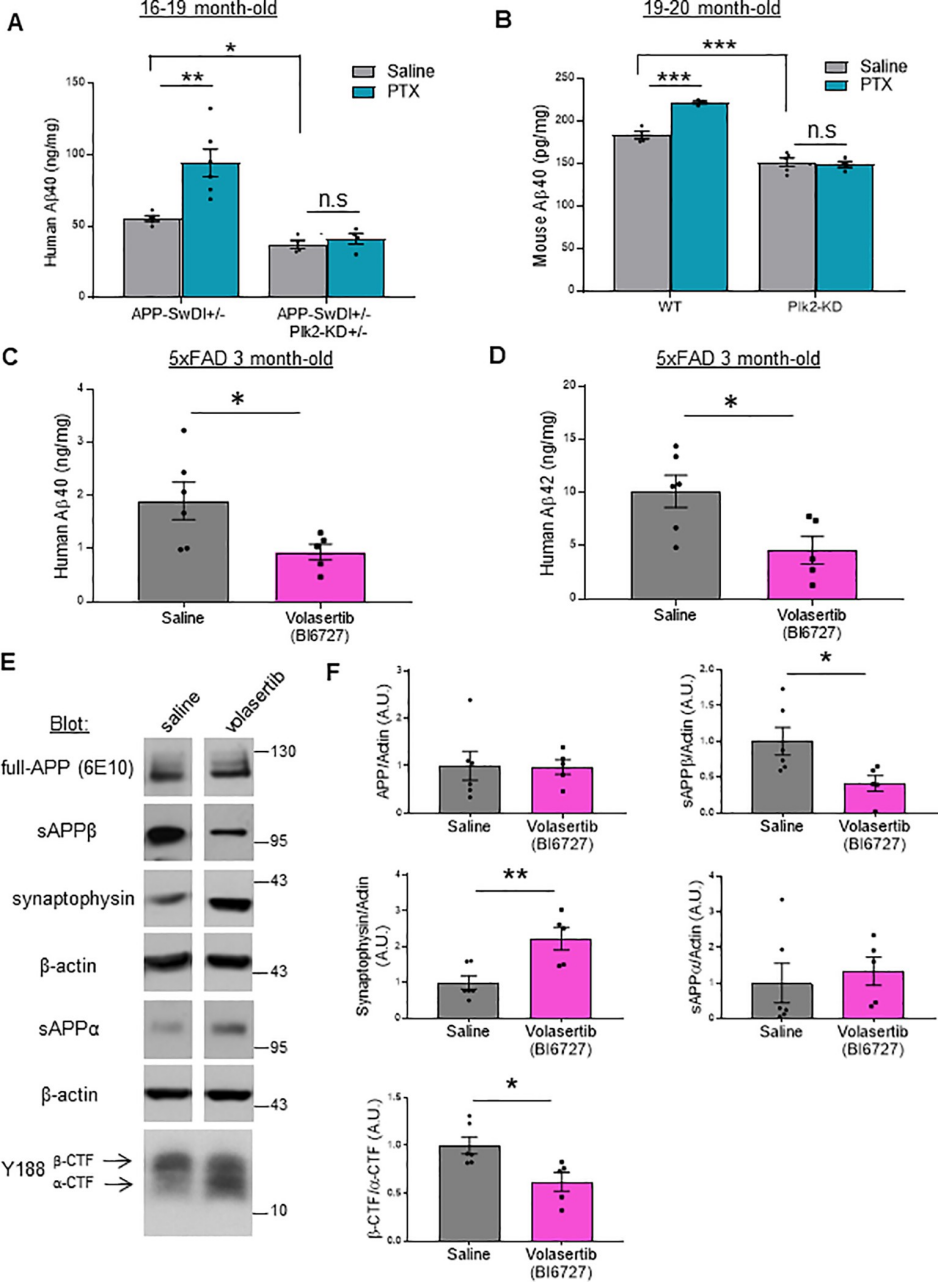
Genetic and pharmacological suppression of Plk2 kinase function reduces APP amyloidgenic processing and Aβ production *in vivo*. (**A, B**) Wild-type (WT), Plk2-KD, APP-SwDI+/-, and APP-SwDI+/-;Plk2-KD+/- mice received daily intraperitoneal injection for 7 days with saline (gray bars) or PTX (1 mg/kg; mint blue bars), and then forebrain Aβ40 measured by species-specific ELISA. (**A**) Quantification of human Aβ40 from 16–19 month-old APP-SwDI+/- and APP-SwDI+/-;Plk2-KD+/- mouse brain (n = 5 animals for saline-treated APP-SwDI+/-, n = 6 for PTX-treated APP-SwDI+/-, and n = 4 for saline- and PTX-treated APP-SwDI+/-;Plk2-KD+/**-**; *F* (3,15) = 17.1, *p* < 0.0001, ANOVA followed by Tukey’s *post hoc* test). (**B**) Quantification of mouse Aβ40 from 19–20 month-old WT or Plk2-KD mice (n = 4 animals for saline- and PTX-treated WT, n = 5 for saline-treated Plk2-KD, and n = 4 for PTX-treated Plk2-KD; *F* (3,13) = 67.16, *p* < 0.0001, ANOVA followed by Tukey’s *post hoc* test). ***p <0.001, **p < 0.01, *p < 0.05, n.s. not significant. (**C, D**) Saline (gray bars) or volasertib (BI6727, 70 mg/kg; pink bars) was administered to 2-month-old 5XFAD male mice every week for a month via oral gavage, followed by measurement of forebrain human Aβ40 (**C**) and Aβ42 (**D**) by ELISA (n = 6 animals for saline, n = 5 for volasertib; Aβ40: *t* (9) = 2.324, *p* = 0.0452; Aβ42: *t* (9) = 2.714, *p* = 0.0238). (**E**) Immunoblot analysis of full length APP, APP cleavage products sAPPβ, sAPPα, and α-/β-CTF, and synaptophysin from saline or volasertib treated 5xFAD mice as indicated; β-actin served as loading controls. Cropped blots are displayed for clarity and concise presentation (full western blots are shown in **S5 Fig**). Molecular weights are in kDa. **(F)** Quantification of **E** (n = 6 animals for saline, n = 5 for volasertib; sAPPβ: *t* (9) = 2.519, *p* = 0.0328; synaptophysin: *t* (9) = 3.436, *p* = 0.0074; α-/β-CTF: *t* (9) = 2.914, *p* = 0.0172). ** *p* <0.01, *p < 0.05, Student’s t-test. Data are means±SEM. Experiments were performed in at least duplicate.

APP-SwDI+/- and APP-SwDI+/-;Plk2-KD+/- hemizygote littermates were further examined for hAβ production. We used Aβ40 to assess amyloidogenic processing for these mice since Plk2 regulates APP processing via BACE-1 [30] and should affect all Aβ variants equally. At 16–19 months of age, forebrain hAβ40 was significantly decreased in APP-SwDI+/-;Plk2-KD+/- mice compared to APP-SwDI+/- littermates, as assayed by human-specific Aβ ELISA (Fig 3A). We also examined endogenous mouse Aβ in 19–20 month-old animals, and we found that Plk2-KD+/- mice had reduced mouse Aβ40 (mAβ40) relative to WT littermates (Fig 3B), suggesting that this regulatory pathway may be relevant for both human and rodent APP processing.

A critical question is whether neuronal activity-stimulated Aβ formation was impaired by Plk2-KD *in vivo* as previously seen in primary cultured neurons [30]. To address this issue, mice received daily intraperitoneal injection for 1 week of either saline or low levels of picrotoxin (PTX, GABA_A_ receptor antagonist) (1 mg/kg). This sub-convulsant threshold dose of PTX administration paradigm has been extensively characterized *in vivo* to model elevated neuronal overactivity within a physiological range [35,47–49] without producing frank seizures. We observed a robust increase in hAβ40 and mAβ40 levels from APP-SwDI+/- and WT mice, respectively, following this mild overactivity challenge, but remarkably, these responses were completely blocked in mice expressing the Plk2-KD transgene (Fig 3A and 3B). Thus, Plk2 was required for activity-regulated induction of Aβ *in vivo*.

To test an independent AD mouse model, we used 5XFAD mice, which preferentially express the more fibrillogenic hAβ42 species and present aggressive pathology manifesting as synapse loss, early onset of plaque deposition, and accelerated memory deficits [39]. The advantage of these mice is that we could test an independent methodology of inhibiting Plk2 kinase function (drug delivery) that has greater translational relevance, and we could obtain results relatively quickly compared to APP-SwDI that develops pathology on a much longer timescale. For this pharmacological approach, we employed the small molecule volasertib (BI6727), originally developed as an anti-cancer drug [36]. This compound is a brain-penetrant Plk family inhibitor that exhibits enhanced pharmacokinetic properties over BI2536, another Plk inhibitor used extensively in prior studies [26,28,29,50]. To validate BI6727 in our system, we examined activity-dependent APP processing in cultured hippocampal neurons. We previously reported that PTX-induced hyperactivity in hippocampal cultured neurons leads to homeostatic downregulation of APP via beta secretase cleavage, and that this process is blocked by BI2536 [30]. As expected, volasertib (BI6727, 50 nM) also prevented the PTX-induced loss of both APP and the postsynaptic marker PSD-95 (S3A–S3D Fig), further supporting the idea that Plks are required for activity-stimulated APP amyloidogenic processing and homeostatic downscaling of excitatory synapses.

Next, for pharmacological *in vivo* studies, volasertib was administered once per week for a month via oral gavage (70 mg/kg) to 5XFAD mice [36]. Interestingly, mice treated with volasertib showed dramatically reduced forebrain levels of both hAβ40 and hAβ42 compared to saline controls (Fig 3C and 3D). Volasertib caused no change in full-length APP, but significantly decreased sAPPβ levels and the β-CTF/α-CTF ratio, along with a trend towards increased sAPPα that did not reach significance (Fig 3E and 3F). Together, these results suggest a decrease in β-processing of APP *in vivo* with volasertib treatment.

To determine whether dampened amyloidogenic processing resulted in reduced pathology in 5XFAD mice, we quantified synaptophysin, a synaptic marker that correlates with dendritic spine and synapse density and is commonly used to assess synapse loss [51]. Synaptophysin levels were significantly greater in volasertib-treated 5XFAD mouse brains compared to saline controls (Fig 3E and 3F). Thus, inhibition of Plk2 kinase function may ameliorate synapse loss in 5XFAD mice.

### Pharmacological inhibition of Plk2 kinase function rescues memory loss

**Fig 4:**
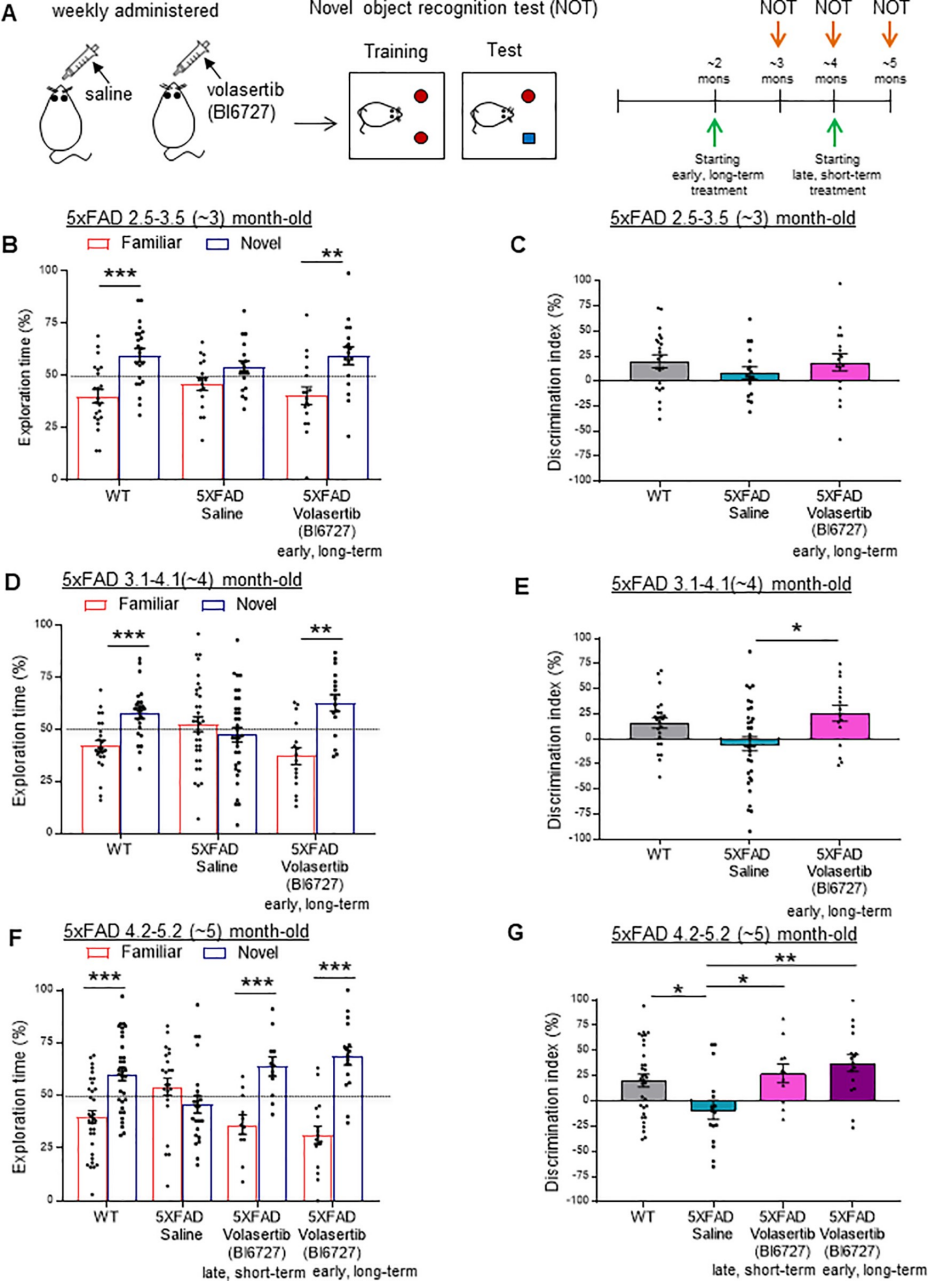
Pharmacological inhibition of Plk2 kinase function rescues memory deficits. (**A**) Schematic of experimental design. Saline or volasertib (BI6727, 70 mg/kg) was administered to 5XFAD male mice every week via oral gavage followed by longitudinal novel object recognition tests (NOT) in 5XFAD and age-matched WT mice. For early (long-term, 3-month) treatment, volasertib was given to 5XFAD mice starting from 1.5–2.5 (~2) months of age. For late (short-term, 1-month) treatment, the drug was given to 5XFAD mice starting from 3.1–4.1 (~4) months of age. Tasks were performed one day after treatment of saline or volasertib. (**B-G**) Percentage of exploration time spent on the familiar object (red) or novel object (blue) (**B,D,F**) or discrimination index (DI) (**C,E,G**) during test sessions with WT and saline- or volasertib-treated 5XFAD mice at 2.5–3.5 (~3) (**B,C**), 3.1–4.1 (~4) (**D,E**), and 4.2–5.2 (~5) (**F,G**) months of age. Discrimination Index was calculated as follows: DI  =  [(Novel Object Exploration Time/Total Exploration Time)—(Familiar Object Exploration Time/Total Exploration Time)] × 100. * *p* <0.05, ** *p* <0.01, and *** *p* <0.001, Student’s t-test (~3 month: WT: *t* (42) = 4.293, *p* = 0.0001, 5XFAD long-term Volasertib (BI6727): *t* (32) = 3.163, *p* = 0.0034; ~4 month: WT: *t* (46) = 4.227, *p* = 0.0001, 5XFAD long-term Volasertib (BI6727): *t* (30) = 4.458, *p* = 0.0001; ~5 month: WT: *t* (64) = 4.693, *p* < 0.0001, 5XFAD short-term Volasertib (BI6727): *t* (20) = 4.254, *p* = 0.0004, 5XFAD long-term Volasertib (BI6727): *t* (30) = 6.239, *p* < 0.0001) (**B,D,F**) or ANOVA with Tukey’s *post hoc* test (~4 month: *F* (2,71) = 4.923, *p* = 0.0099; ~5 month: *F* (3,72) = 5.137, *p* = 0.0028) (**C,E,G**); n = 33 for WT, 23 for saline, 11 for late, and 16 animals for early treatment. Data are means±SEM.

To confirm whether this blockade of APP amyloidogenic processing by volasertib also led to improvement of cognitive dysfunction, we administered volasertib or saline once per week and performed longitudinal novel object recognition tests (NOT) in 5XFAD male mice as well as age-matched WT male mice (Fig 4A, left). We divided the volasertib-treated 5XFAD mice into two groups (early and late) in order to identify an ideal intervention time for treatment. The early group received a long-term 3-month treatment starting from 1.5–2.5 months old (~2 month cohort) when plaque deposits begin to initially form, whereas the late group was given a short-term 1-month treatment starting from 3.1–4.1 months of age (~4 month cohort) when memory deficits have already manifested [39,52,53] (see experimental scheme in Fig 4A, right). As a control, we confirmed that none of the groups showed any inherent preference for right or left side of the testing chamber during familiarization (training) sessions (S4 Fig).

Between ~3–5 months of age, WT mice spent significantly more time in exploring a novel object than a familiar one, as expected, but saline-treated 5XFAD mice showed no preference, indicating a failure of memory in the latter group (Fig 4B, 4D and 4F). However, volasertib treatment rescued this deficit in 5XFAD mice in the early, long-term treatment group at all time points tested, as measured by restoration of exploratory preference for novel objects (Fig 4B, 4D and 4F). The ability to distinguish between novel and familiar objects was also quantified by the discrimination index (difference in percentage of time spent between novel and familiar objects). WT and volasertib-treated 5XFAD early group mice showed a significant difference in discrimination index compared to saline-treated 5XFAD mice at 5 months of age, but not at earlier time points (Fig 4C, 4E and 4G).

Notably, recognition memory was also rescued in the late, short-term volasertib treatment group, which displayed a higher preference for novel over familiar objects and a significant difference in discrimination index compared to the saline-treated 5XFAD mice (Fig 4F and 4G). This finding implies that volasertib may provide cognitive benefits even after memory deficits have begun to develop.

Thus, we conclude that Plk2 contributes substantially to localized amyloid plaque formation and mediates physiological activity-dependent amyloidogenic APP processing in the brain, and that suppressing this pathway can functionally rescue disease-relevant synaptic pathology and memory impairment.

## Discussion

In this study, we examined the potential role of Plk2 in AD pathogenesis using two different mouse models as well as human AD brain tissue, building on our previous *in vitro* finding that Plk2 kinase function stimulates APP amyloidogenic processing [30]. A role for Plk2 in promoting amyloidogenic processing implies elevated Plk2 levels in AD. Indeed, as previously reported [33], we confirmed that Plk2 was upregulated in human AD entorhinal cortex and hippocampus, which are the brain areas most afflicted in the disease and show heaviest accumulation of plaques and tangles [3,43,44]. We further showed using within-subject controls that Plk2 was not upregulated in cerebellum or occipital lobe, brain regions largely spared in AD. Thus, Plk2 upregulation correlates with regional disease severity in human AD brain.

Plk2 induction was also observed in a transgenic mouse model of AD in a spatiotemporally controlled manner. We examined Plk2 expression and plaque load by immunohistochemistry at different ages in the cortex and hippocampal regions in APP-SwDI mice, and we found that in the early phase of plaque formation (at 4 months of age), Plk2 was upregulated in both hippocampus and cortex. In older APP-SwDI mice (7 months) during the period of robust Aβ plaque deposition, Plk2 levels remained elevated in cortex, but no longer in the hippocampus. These findings suggest that Plk2 is differentially induced in various brain regions over time. Additionally, increased Plk2 levels were spatially coincident with the distribution of plaques, and Plk2 was preferentially clustered near large amyloid deposits. These results support a model in which aberrant Plk2 expression, which is known to be synaptic activity-inducible, may be driven by local pockets of neuronal hyperactivity that exist in the vicinity of amyloid plaques in several mouse AD models [54,55]. Taken together with the finding that Plk2 in turn promotes amyloidogenic processing, this situation would result in a positive feedback loop of ever-increasing amyloid production and Plk2 induction.

To interfere with this potential vicious cycle of pathogenesis *in vivo*, we crossed APP-SwDI and dominant negative Plk2-KD transgenic mice. In the forebrain of double hemizygous progeny, we measured significant reduction in basal hAβ load and activity-induced hAβ production. Furthermore, Plk-KD reduced endogenous rodent Aβ levels compared to WT littermates as well. These results suggest that blocking Plk2 kinase function reduces APP amyloidogenic processing *in vivo*. Since Plk2 only affects activity-dependent Aβ production and not constitutive synthesis, these findings demonstrate for the first time that this inducible pathway significantly contributes to plaque deposition.

It is notable that the decrease in Aβ and plaques due to Plk2-KD was modest under basal (non-stimulated) conditions. This observation may reflect the fact that the Plk2-KD transgene expression only partially overlaps with APP-SwDI, and has even less overlap with endogenous APP. Plk2-KD is expressed via the CaMKIIα promoter only in forebrain [28], whereas APP-SwDI is expressed in cortex, hippocampus, olfactory bulb, cerebellum and thalamus [42]. A significant amount of Aβ could also arise from APP expressed in other neuron types or (in the case of endogenous APP) from astrocytes.

Because Plk2 is induced in response to intense neuronal activation, it may be that only strong forms of synaptic activity will produce Aβ effectively. Such neuronal hyperactivity should not be widely prevalent in WT mice, but may occur sporadically in hot spots, and this gradual stimulation of Aβ may be related to the long development of the disease in sporadic AD. The effects of Plk2 inhibition should therefore be more pronounced under conditions of elevated neuronal activation. Previously, when we maximally stimulated *in vitro* neuron cultures with high concentrations of PTX to produce strong hyperexcitation, the effect on synaptic APP processing and subsequent rescue by Plk2 inhibition was most dramatic [30]. *In vivo*, we used a milder activity paradigm with low-dose PTX to approximate more physiological conditions of heightened activity rather than extreme seizure conditions, which may be less relevant for AD. As expected, with activity stimulation we observed a larger reduction in Aβ with Plk2-KD–indeed, the induction of Aβ was abolished, indicating that Plk2 is essential for this process. Furthermore, inhibition of Plk2 function in mice with the Plk2-KD transgene would be expected to produce increased hyperactivity in response to PTX compared to APP-SwDI+/- mice, because one mechanism of homeostatic downregulation (mediated by Plk2) is impaired. Thus, the lower Aβ production in APP-SwDI+/-;Plk2-KD+/- mice is not likely attributable to a reduced response to PTX in these animals.

Finally, using a pharmacological approach with volasertib we observed a more pronounced effect in reducing Aβ (both Aβ40 and Aβ42) in 5xFAD mice even under basal conditions, possibly because the systemic drug affects APP more widely than the regionally restricted Plk2-KD transgene could. Moreover, it may be that even under basal conditions there is a high level of hyperactivity and therefore endogenous Plk2 in 5xFAD mice, consistent with the overproduction of exogenous Aβ in these animals and the known Aβ-dependent neuronal hyperexcitation in 5xFAD and other AD models [18,54,56]. Volasertib also rescued 5xFAD mice from synapse loss, potentially slowing the progression of cognitive impairment [57–59]. Indeed, the volasertib-treated 5XFAD mice strongly favored novel objects in the novel object recognition task, similar in performance to WT mice, whereas the saline-treated 5XFAD mice did not. These results suggest that the drug rescues memory deficits in AD mice. Since volasertib is currently in phase 3 clinical trials for cancer treatment [60,61], this drug could potentially be repurposed for AD. However, we should be cautious with the interpretation of this experiment because volasertib is a pan-specific Plk family inhibitor and has dose-dependent side effects including hair loss [60]. Further studies with compounds more selective to Plk2 may exhibit greater efficacy and lower toxicity. We did not test WT mice with volasertib because the drug by itself in hippocampal cultures did not cause any change in the levels of PSD-95, a postsynaptic marker highly associated with learning and memory [62–64]. Therefore, we speculate that administration of volasertib may not affect memory in WT mice. However, we cannot rule out a potential effect of the drug on cognition in normal animals, which would be of interest to determine in future studies.

Taken together, we propose that Plk2 and APP are involved in a homeostatic negative feedback loop, physiologically acting to dampen overactivity in neurons. Plk2 induced by synaptic overactivity binds directly to the intracellular tail of APP and phosphorylates residues T668/S675, thus triggering APP endocytosis and Aβ formation [30]. We speculate that this mechanism may play a part in the negative feedback homeostasis to prevent overexcitation, but further investigation will be necessary to establish this possibility. During pathogenesis, aberrant Aβ aggregation may undermine the physiological homeostatic system, generating oligomer-mediated hyperexcitability [18,54,55,65]. In this circumstance, a positive feedback loop occurs and promotes further aggregates and foci of synaptic overactivity, as we observed in the APP-SwDI brain where elevated Plk2 was localized in the vicinity of actively depositing plaques. Plk2 upregulation is thus a “snapshot” of local hotspots of dysfunction in neuronal homeostasis, whereas visible plaque deposits stand for a cumulative readout of disease progression.

It is interesting to note that both Aβ and Plk2 cause synapse loss, a process which is strongly associated with cognitive impairment in AD [3], while pharmacological inhibition of Plk2 function ameliorates synapse loss and memory decline in an aggressive mouse model of AD. Our proposed pathway may provide novel therapeutics for synapse loss and memory impairments in AD by engaging physiological regulatory programs in the control of APP metabolism.

## Supporting information

S1 Fig Plk2 levels and neuronal expression in brain. (**A,B**) Perfused brain sections from *APP-SwDI*^*+/+*^ (**A**) and cryosections from APP-*SwDI*^*+/-*^*;Plk2-KD*^*+/-*^ (**B**) mice were immunolabeled with human Aβ antibody 6E10 (magenta) and counterstained with thioflavin S (green) demonstrating high degree of overlap. Scale bar, 100 μm. (**C**) Perfused section from WT and cryosection from *Plk2-KD*^*+/-*^ brains stained with thioflavin S lack detectable plaques. (**D**) Cresyl violet-stained coronal section of mouse brain illustrating analyzed regions (Rs, retrosplenial cortex; PtA, parietal association cortex; S1Tr, primary somatosensory cortex; S1BF, primary somatosensory cortex, barrel field; S2 secondary somatosensory cortex; AuV, secondary auditory cortex; DG, dentate gyrus; CA1 and 3, cornu ammonis subregions of hippocampus). (**E**) Representative sections of primary somatosensory cortex and hippocampus from 4-month-old WT and *APP-SwDI*^*+/+*^ mice stained with thioflavin S (green) and immunolabeled with Plk2 antibody C-18 (magenta). (**F,G**) Quantification of (**F**) number of Plk2 expressing neurons and (**G**) Plk2 integrated intensity at 4 months in WT and *APP-SwDI*^*+/+*^ transgenic (TG) mice (n = 4–14 regions from 4 animals). (**H**) Plaque load (% of area) in *APP-SwDI*^*+/+*^ cortical and hippocampal regions at 4 and 7 months (n = 8–12 regions from 4 animals for 4 months and n = 16–22 regions from 6 animals for 7 months). (**I**) Representative cerebellar (CRBL) or occipital lobe (OCC) sections from control and AD samples of postmortem human brains immunolabeled with Plk2 antibody H-90. Scale bar, 100 μm. (**J**) Quantification of **I** (n = 5 cases for control and 5 for AD; ns, not significant vs. control). (**K,L**) Representative hippocampal CA1 section of *APP-SwDI*^*+/+*^ mouse brain immunolabeled with 6E10 and Plk2 antibody C-18 (**K**) and in human AD brain with Plk2 antibody H-90 (**L**) showing enriched Plk2 expression in neurons of stratum pyramidale. Right, high magnification views of boxed areas. Scale bars: wide field, 50 μm (**K**) and 100 μm (**L**); higher magnification, 10 μm. (**M,N**) Representative cortical sections of WT and *APP-SwDI*^*+/+*^ mouse (**M**) and human control or AD brain (**N**) stained with only secondary antibodies as control for background staining; scale bars, 50 μm (**M**) and 100 μm (**N**). ***p < 0.001, **p < 0.01, *p < 0.05; two-tailed unpaired Student’s t-test. Data are means±SEM. Experiments were performed in at least duplicate.
(PDF)

S2 Fig Plaque level is unchanged in *APP-SwDI* mouse brain regions lacking Plk2-KD expression. (**A**) Representative thalamus sections of *APP-SwDI+/-* and *APP-SwDI+/-;Plk2-KD+/-* mice stained with thioflavin S. Po, posterior nucleus of thalamus; VPM, ventral posteromedial nuclus; VPL, ventral posterolateral nucleus. (**B**) Quantification of plaque area in thalamic subregions (n = 10 regions from 5 animals for APP-SwDI+/- and n = 6 regions from 6 animals for *APP-SwDI+/-;Plk2-KD+/-*). Data are means±SEM. Experiments were performed in at least duplicate.
(PDF)

S3 Fig Activity-dependent loss of APP and postsynapses requires Plks in cultured hippocampal neurons. (**A,B**) Representative dendrites of rat primary hippocampal neurons (DIV 20–24) treated with picrotoxin (PTX, 25 μM, 18–20 h) or vehicle (NT) and co-treated with different doses of Plk inhibitor BI6727 (volasertib) as indicated, then co-immunolabeled with APP-N antibody and PSD-95. Complete block of activity-dependent APP processing was achieved at 50 nM BI6727. (**C**) Quantification of **A** (total APP intensity). (**D**) Quantification of **B (**PSD-95 intensity) (n = 36 neurons for NT, 43 for BI6727 5nM, 35 for BI6727 10nM, 30 for BI6727 50nM, 30 for PTX, 48 for PTX+BI6727 5 nM, 40 for PTX+BI6727 10 nM, 32 for PTX+BI6727 50 nM). ***p < 0.001; **p < 0.01, *p < 0.05; ANOVA with Tukey’s *post hoc* test. Data are means±SEM. Experiments were performed in at least duplicate.
(PDF)

S4 Fig Mice show no inherent location preference in novel object testing chamber. 5XFAD mice were given saline or volasertib (BI6727, 70 mg/kg) weekly via oral gavage followed by novel object recognition tests (NOT) with age-matched WT mice. See experimental design in **Fig 4A**. Percentage of exploration time spent in left (red) or right (blue) object during initial training sessions with WT and saline- or volasertib-treated 5XFAD mice (n = 33 for WT, n = 23 for saline, n = 11 for late volasertib treatment, and n = 16 for early volasertib treatment). Data are means±SEM. Experiments were performed in at least duplicate.
(PDF)

S5 Fig Uncropped western blots. Regions of blots used for main figures are shown boxed in red. Molecular weights are in kDa.
(PDF)

S1 Table Demographic and pathological characteristics of AD and control cases. (PDF)
