# Supplementary figures and images for: Inhibition of Polo-like kinase 2 ameliorates pathogenesis in Alzheimer’s disease model mice
Source: PLoS One. 2019 Jul 15;14(7):e0219691. doi: 10.1371/journal.pone.0219691 (PMC6629081; doi:10.1371/journal.pone.0219691)

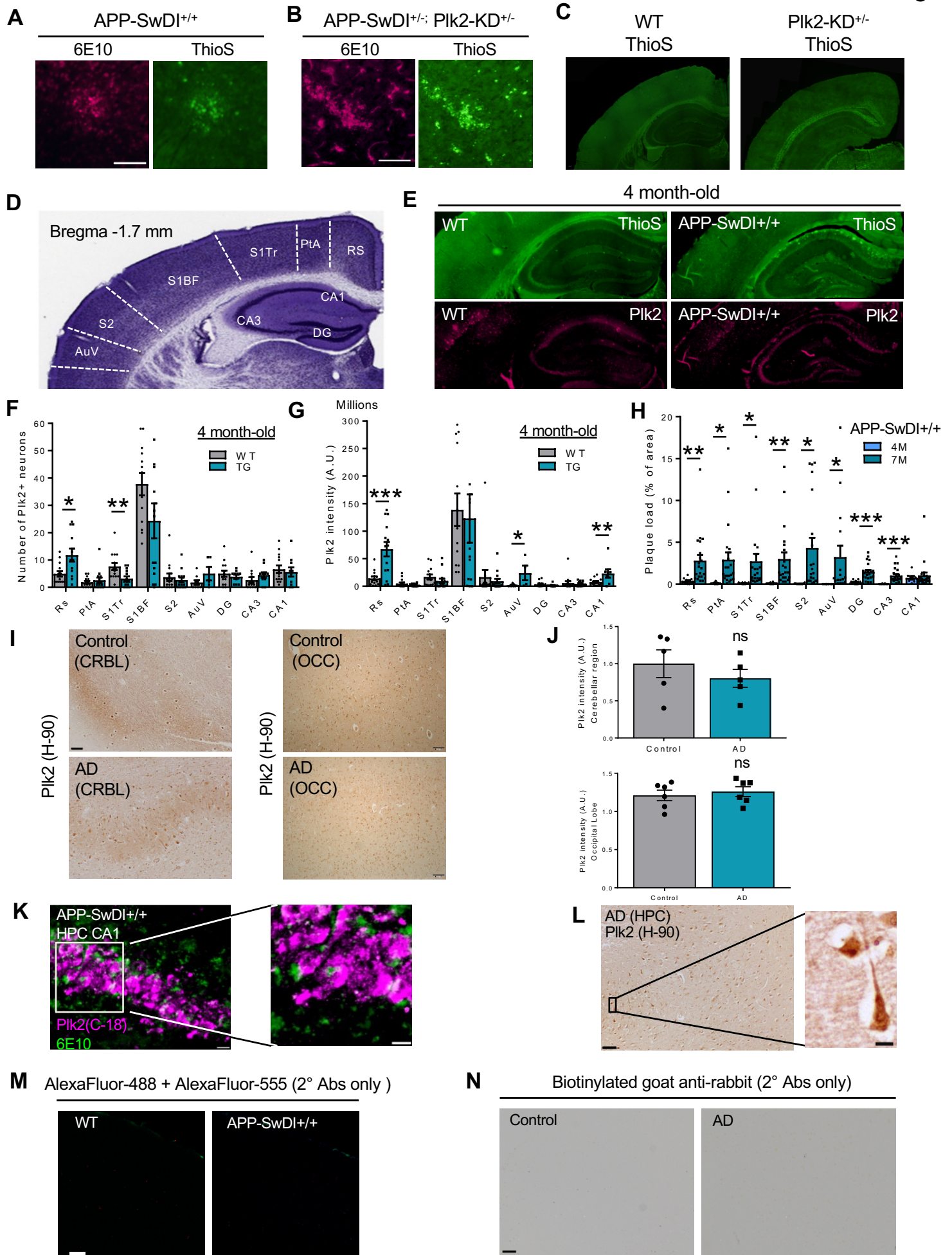

Supplement: S1 Fig — (A,B) Perfused brain sections from APP-SwDI+/+ (A) and cryosections from APP-SwDI+/-;Plk2-KD+/- (B) mice were immunolabeled with human Aβ antibody 6E10 (magenta) and counterstained with thioflavin S (green) demonstrating high degree of overlap. Scale bar, 100 μm. (C) Perfused section from WT and cryosection from Plk2-KD+/- brains stained with thioflavin S lack detectable plaques. (D) Cresyl violet-stained coronal section of mouse brain illustrating analyzed regions (Rs, retrosplenial cortex; PtA, parietal association cortex; S1Tr, primary somatosensory cortex; S1BF, primary somatosensory cortex, barrel field; S2 secondary somatosensory cortex; AuV, secondary auditory cortex; DG, dentate gyrus; CA1 and 3, cornu ammonis subregions of hippocampus). (E) Representative sections of primary somatosensory cortex and hippocampus from 4-month-old WT and APP-SwDI+/+ mice stained with thioflavin S (green) and immunolabeled with Plk2 antibody C-18 (magenta). (F,G) Quantification of (F) number of Plk2 expressing neurons and (G) Plk2 integrated intensity at 4 months in WT and APP-SwDI+/+ transgenic (TG) mice (n = 4–14 regions from 4 animals). (H) Plaque load (% of area) in APP-SwDI+/+ cortical and hippocampal regions at 4 and 7 months (n = 8–12 regions from 4 animals for 4 months and n = 16–22 regions from 6 animals for 7 months). (I) Representative cerebellar (CRBL) or occipital lobe (OCC) sections from control and AD samples of postmortem human brains immunolabeled with Plk2 antibody H-90. Scale bar, 100 μm. (J) Quantification of I (n = 5 cases for control and 5 for AD; ns, not significant vs. control). (K,L) Representative hippocampal CA1 section of APP-SwDI+/+ mouse brain immunolabeled with 6E10 and Plk2 antibody C-18 (K) and in human AD brain with Plk2 antibody H-90 (L) showing enriched Plk2 expression in neurons of stratum pyramidale. Right, high magnification views of boxed areas. Scale bars: wide field, 50 μm (K) and 100 μm (L); higher magnification, 10 μm. (M,N) Represent [file pone.0219691.s001.pdf]

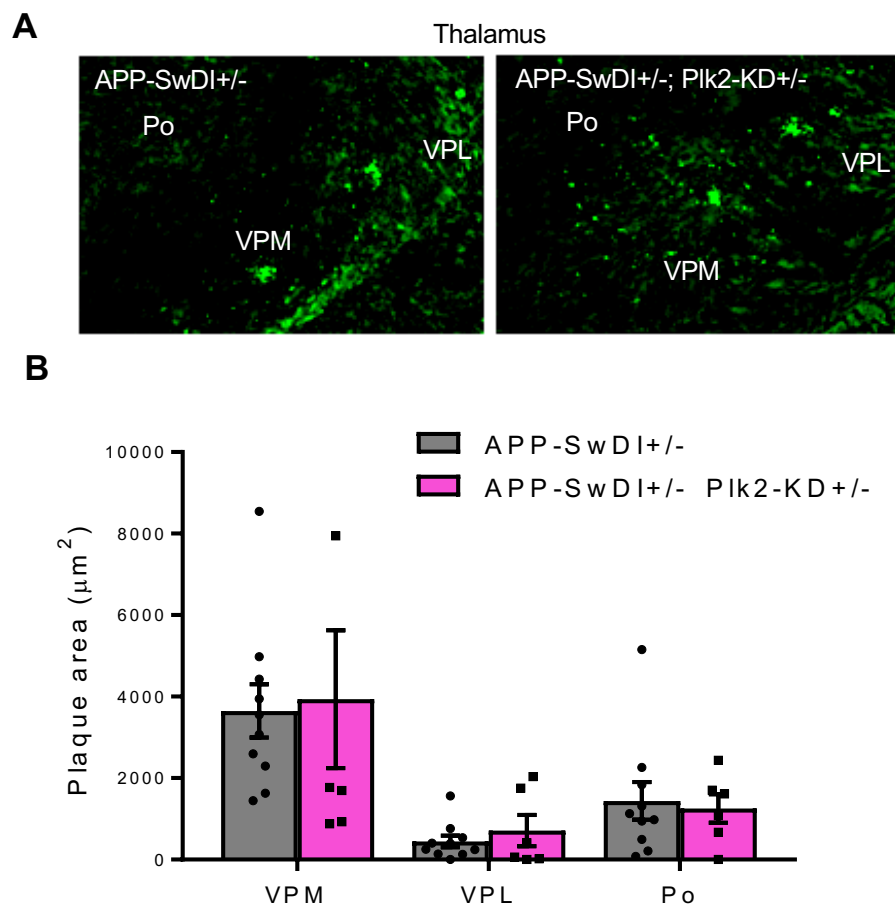

Supplement: S2 Fig — (A) Representative thalamus sections of APP-SwDI+/- and APP-SwDI+/-;Plk2-KD+/- mice stained with thioflavin S. Po, posterior nucleus of thalamus; VPM, ventral posteromedial nuclus; VPL, ventral posterolateral nucleus. (B) Quantification of plaque area in thalamic subregions (n = 10 regions from 5 animals for APP-SwDI+/- and n = 6 regions from 6 animals for APP-SwDI+/-;Plk2-KD+/-). Data are means±SEM. Experiments were performed in at least duplicate. (PDF) [file pone.0219691.s002.pdf]

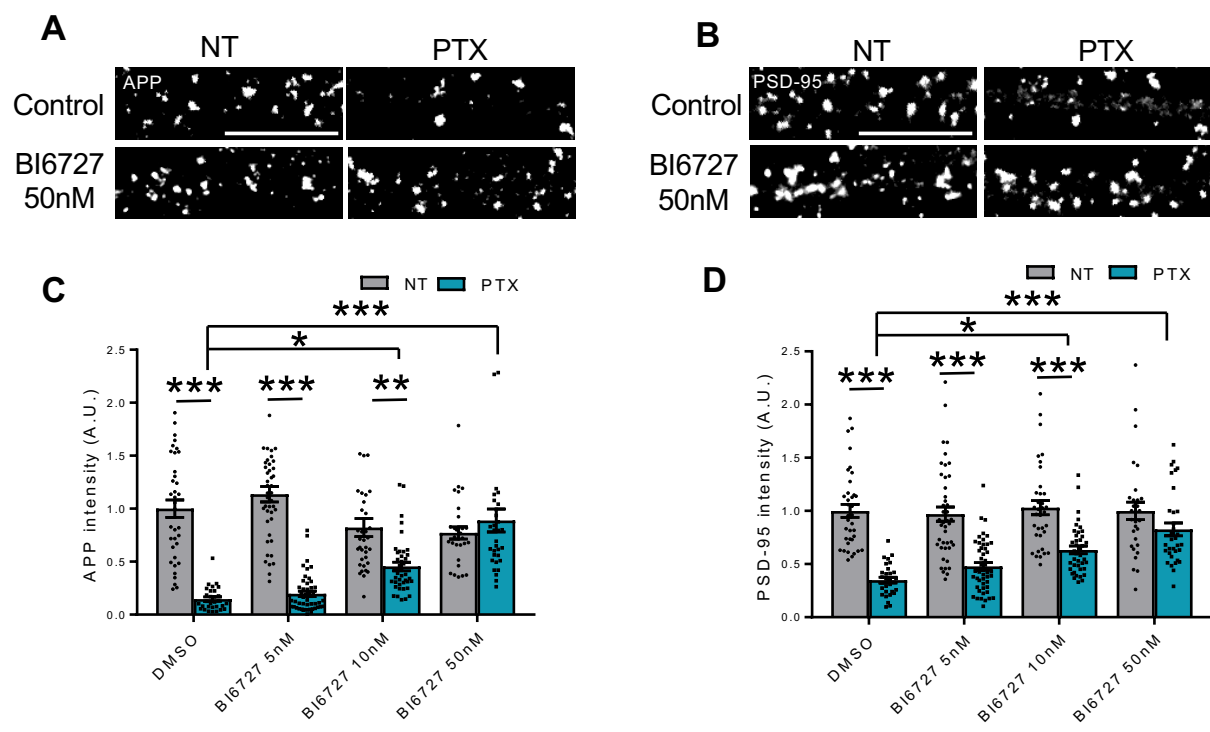

Supplement: S3 Fig — (A,B) Representative dendrites of rat primary hippocampal neurons (DIV 20–24) treated with picrotoxin (PTX, 25 μM, 18–20 h) or vehicle (NT) and co-treated with different doses of Plk inhibitor BI6727 (volasertib) as indicated, then co-immunolabeled with APP-N antibody and PSD-95. Complete block of activity-dependent APP processing was achieved at 50 nM BI6727. (C) Quantification of A (total APP intensity). (D) Quantification of B (PSD-95 intensity) (n = 36 neurons for NT, 43 for BI6727 5nM, 35 for BI6727 10nM, 30 for BI6727 50nM, 30 for PTX, 48 for PTX+BI6727 5 nM, 40 for PTX+BI6727 10 nM, 32 for PTX+BI6727 50 nM). ***p < 0.001; **p < 0.01, *p < 0.05; ANOVA with Tukey’s post hoc test. Data are means±SEM. Experiments were performed in at least duplicate. (PDF) [file pone.0219691.s003.pdf]

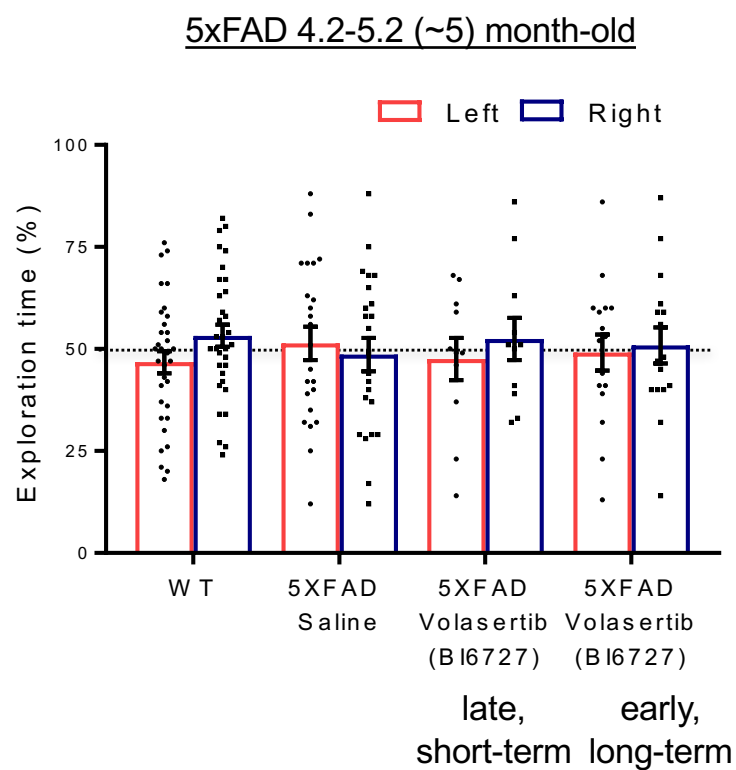

Supplement: S4 Fig — 5XFAD mice were given saline or volasertib (BI6727, 70 mg/kg) weekly via oral gavage followed by novel object recognition tests (NOT) with age-matched WT mice. See experimental design in Fig 4A. Percentage of exploration time spent in left (red) or right (blue) object during initial training sessions with WT and saline- or volasertib-treated 5XFAD mice (n = 33 for WT, n = 23 for saline, n = 11 for late volasertib treatment, and n = 16 for early volasertib treatment). Data are means±SEM. Experiments were performed in at least duplicate. (PDF) [file pone.0219691.s004.pdf]

Fig. 3E-F

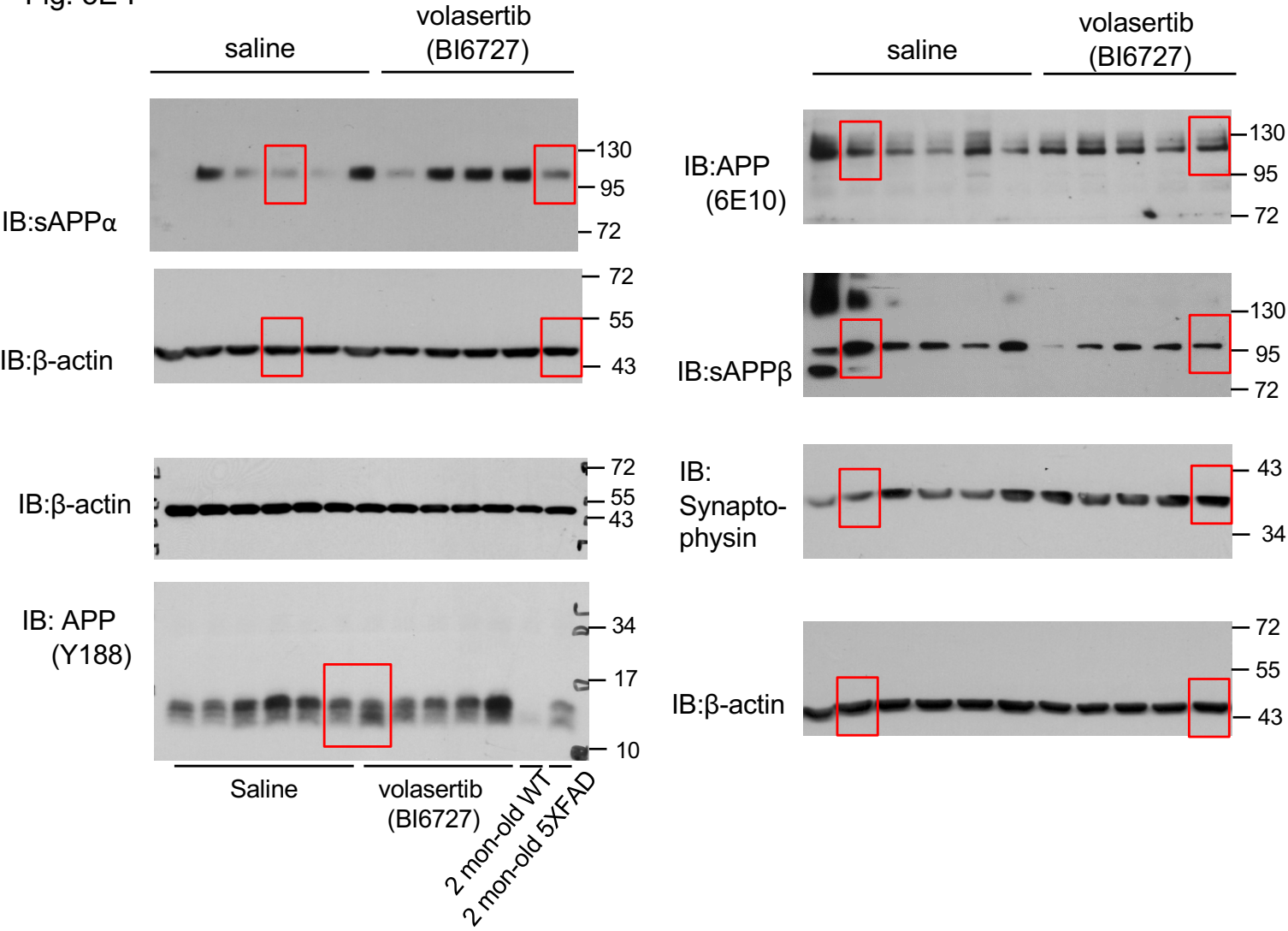

Supplement: S5 Fig — Regions of blots used for main figures are shown boxed in red. Molecular weights are in kDa. (PDF) [file pone.0219691.s005.pdf]
