# Supplementary material for: Inhibition of Polo-like kinase 2 ameliorates pathogenesis in Alzheimer’s disease model mice
Source: PLoS One. 2019 Jul 15;14(7):e0219691. doi: 10.1371/journal.pone.0219691 (PMC6629081; doi:10.1371/journal.pone.0219691)
Supplement: S1 Table — (PDF) [file pone.0219691.s006.pdf]

Supplementary Table 1. Demographic and pathological characteristics of AD and control cases

Case no.	Age (years)	Gender	ABC score			Other neuropathies
			Amyloid	Braak	CERAD	
Control 1	61	M	NA	NA	NA	Moderate hypertensive vasculopathy, Diffuse acute hypoxic ischemic injury
Control 2	99	F	NA	NA	NA	Moderate hypertensive vasculopathy
Control 3	74	M	NA	NA	NA	CNS metastatic melanoma, Hypertensive vasculopathy, diffuse acute hypoxic-ischemic injury
Control 4	72	M	NA	NA	NA	Mild atherosclerosis and hypertensive vasculopathy, metabolic glia
Control 5	41	M	NA	NA	NA	Mild acute hypoxic ischemic injury
Mean ± S.E.M	69.4 ± 9.4	F/M 1/4				
AD 1	104	F	A3	B3	C2	Severe atherosclerosis, Severe Hypertensive Vasculopathy, Mild amyloid angiopathy
AD 2	67	F	A2	B3	C2	Mild amyloid angiopathy
AD 3	77	F	A3	B3	C3	Status post ventriculoperitoneal (VP) shunt
AD 4	74	F	A3	B3	C3	Hypertensive vasculopahy
AD 5	81	F	A1	B2	C1	Multifocal actute/subacute hemorrhagic infacts, Moderate hypertensive vasculopathy, status post VP shunt
AD 6	97	M	A2	B3	C2	Severe hypertensive vasculopathy, Moderate amyloid angiopathy, remote frontal infarct
Mean ± S.E.M	83.3 ± 5.8	F/M 5/1				

ABC score: amyloid plaque deposits (Amyloid score: A0 (not), A1 (low), A2 (intermediate), A3 (high)), neurofibrillary tangle (Braak stage: B0 (not), B1 (low), B2 (intermediate), B3 (high)), and neuritic plaque density (CERAD (Consortium to Establish a Registry for Alzheimer’s Disease) score : C0 (none), C1 (sparse), C2 (moderate), C3 (frequent)); NA, not applicable
